# Effect of phenyllactic acid on silage fermentation and bacterial community of reed canary grass on the Qinghai Tibetan Plateau

**DOI:** 10.1186/s12866-022-02499-w

**Published:** 2022-03-30

**Authors:** Yongxiang Lu, Ping Li, Shiqie Bai, Shiyong Chen, Man Zhao, Wenlong Gou, Minghong You, Qiming Cheng

**Affiliations:** 1 Department of Grassland Science, Guizhou University, Guiyang, 550025 China; 2 Sichuan Academy of Grassland Science, Chengdu, 611731 China; 3 Institute of Qinghai-Tibet Plateau Research, Southwest Minzu University, Chengdu, 610041 China

**Keywords:** Bioactive agents, *Phalaris arundinacea*, Ensilage, Fermentation quality, Bacterial population

## Abstract

**Background:**

This study aimed to investigate the effect of phenyllactic acid as an additive on silage fermentation and bacterial community of reed canary grass (RCG, *Phalaris arundinacea* L.) on the Qinghai Tibetan Plateau. At the heading stage, RCG was harvested, chopped and ensiled in small bag silos. The silage was treated without (control, 1.0 g/mL sterile water, on a fresh matter basis (FM)) or with phenyllactic acid (PLA, 3 mg/mL, FM), antimicrobial additive (PSB, a mixture of potassium sorbate and sodium benzoate, 2%, FM), lactic acid bacteria inoculant (LABi, *L. plantarum* + *L. curvatus*, 1 × 10^6^ cfu/g, FM) and PLA + LABi, and then stored in a dark room at the ambient temperature (5 ~ 15 °C) for 60 days.

**Results:**

Compared with control, PLA decreased lactic acid, acetic acid and ammonia-N contents, and subsequently increased CP content of RCG silage. PLA enhanced the growth of lactic acid bacteria and reduced the count of yeasts (*P* < 0.05) in RCG silage, with reduced bacterial richness index (Chao1), observed operational taxonomic units and diversity index (Simpson). In relative to control, moreover, PLA and PLA + LABi increased the relative abundance of *Lactococcus* in RCG silage by 27.73 and 16.93%, respectively.

**Conclusions:**

Therefore, phenyllactic acid at ensiling improved nutritional quality of RCG silage by advancing the disappearance of yeasts and the dominance of *Lactococcus*.

## Background

The Qinghai-Tibetan Plateau (QTP) is an important alpine grassland livestock production region [1]. However, the unstable weather conditions result in forage shortage for herbivores in winter and early spring [2], which cause yaks’ weight loss, low milk production and other problems [3]. Therefore, how to preserve local forages for enhancing milk production and meat quality of yaks is a concerned issue [4].

Ensiling is a good way to preserve the nutritional value of forages, especially in areas where forages are seasonally or regionally unbalanced due to the harsh environment. In cold regions, silage is considered to be a main source of feed for ruminants [5]. Recently, Chen et al. and Zhao et al. have reported that chemical and microbial additives can improve the quality of silage on the Qinghai Tibetan Plateau [6, 7]. However, silage also faces challenges in cold regions. For example, silage exposed to the air is easy to deteriorate, and some desirable inherent/exogenous microbes (mainly lactic acid bacteria, LAB) are scarce in a low temperature environment [8, 9]. In addition, yeasts and other harmful microorganisms showed a higher activity in silages under cool/cold conditions [10]. These reduce the positive effect from additives on preservation of silage nutrients. Importantly, some studies have reported that the use of some conventional chemical additives such as formic acid can cause different health problems to people [11]. Therefore, it is necessary to explore new alternatives to stabilize silage quality for safe production of milk and meat in cold regions.

Phenyllactic acid (PLA) is a kind of small molecule organic acid, which exists widely in the nature and is harmless to humans and animals [12–14]. Rvr et al. reported that PLA has been used as a substitute for antibiotics in livestock feed and dairy products, with a good effect on extending the storage period of dairy products and improving the quality of meat products [11]. However, there is little information on PLA-treated silage [15].

The reed canary grass (RCG, *Phalaris arundinacea* L.) is a kind of promising high-yield cool-season forage. It can tolerate different environmental conditions, including cold and moist conditions. It also had a high-yield potential during the harvest season. Despite of many reports about RCG silage on the QTP [16–18], there is still little information on PLA-treated RCG silage. Therefore, an experiment was designed to investigate the effect of PLA on the fermentation quality and bacterial community of RCG silage on the QTP. We supposed that (1) PLA can improve the fermentation quality of reed canary grass silage. (2) PLA can change the diversity of the bacterial community and the composition of the recombinant bacterial community.

## Methods

### Silage preparation

The experiment was conducted at the experiment base of Sichuan Academy of Grassland Science (N 31°51′-33°33′, E 101°51′-103°22′, altitude 3500 m; Hongyuan, Sichuan, P.R. China). RCG (*P. arundinacea* L. ‘Chuancaoyin No.3’ cultivated by Sichuan Academy of Grassland Sciences) at the heading stage was harvested manually, and chopped by the length of 1–3 cm. According to previous report from Chen et al., pre-ensiled RCG featured water soluble carbohydrates (WSC) of 7.88%DM, crude protein (CP) of 12.91%DM, neutral detergent fiber (NDF) 53.96%DM and acid detergent fiber (ADF) of 30.92%DM, lactic acid bacteria (LAB) of < 3.0 log_10_cfu/g FM and yeast and mold of > 4.0 log_10_cfu/g FM [18]. The chopped RCG was randomly divided into five equal parts for the following treatments: no additives (control); lactic acid bacteria inoculant (LABi, a mixture of *L. plantarum* and *L. buchneri*, each 10^6^ cfu/g fresh matter (FM) base); phenyllactic acid (PLA, 3 mg/kg FM); antimicrobial additive (PSB, a mixture of potassium sorbate and sodium benzoate, each 1.0% FM); and PLA+ LABi. Each treatment was repeated for three times. About 300 g of the mixed-RCG was put into 30 cm × 25 cm polyethylene bag, vacuumed and sealed by a vacuum sealer, and stored at ambient temperature (5 ~ 15 °C) for 60 days.

### Chemical analysis

The silage samples were dried at 65 °C for a constant weight to determine the content of DM [19]. And then they were ground with a 1 mm sieve for other nutrient analysis. The CP was determined by the method of Kjeldahl. Both NDF and ADF were determined using an Ankom 2000 fiber analyzer (Ankom Technology, Fairport, NY). The WSC was measured by the method of Murphy [20], weighed 0.2 g of the crushed sample and placed it in a test tube, added 10 mL distilled water and boiled for 30 min, filtered in a 25 mL volumetric flask, pipetted 1 mL of the above sugar extract, put into a test tube, added 5 mL anthrone solution, and boiled for 10 min, took out to cool, then measured the absorbance value (OD) at the 620 nm wavelength, used the standard curve to calculate the sugar extract content, finally, obtained the soluble sugar content by formula.

$$\mathrm{WSC}\ \left(\%/\mathrm{DM}\right)=\mathrm{A}\ast \mathrm{C}/\left(\mathrm{W}\ast 1{0}^4\right)$$

Note: A represents the volume of extraction liquid.

C represents the sugar content in extract (ug/mL).

W represents the sample weight (g).

(the standard curve line y = 0.0075x + 0.0665 R^2^ = 0.9984 y: OD x = C)

Fresh sample (20 g) was mixed with 180 mL ultrapure water for 3 min in a Stomacher blender. The pH of filtrate was determined by a pH meter. About 10 mL was filtrated by centrifugation (4500×g, 15 min, 4 °C), and the supernatant was analyzed for contents of lactic acid (LA), acetic acid (AA), propionic acid (PA) and butyric acid (BA) by high performance liquid chromatography [21] (Color spectrum column: Shodex Rspak KC-811 S-DVB gel column, Detector: SPD-M10AVP, the mobile phase used: 3 mmol/L high chloric acid solution, the set flow rate: 1 mL/min, the column temperature: 50 °C, the detection wave: 210 nm, Sample volume: 5 μL, injection time: 25 min). Ammonia nitrogen (AN) was determined by the methods of Broderick and Kang [22], took 1 mL filtered sample solution, added 4 mL of 0.2 mol/L hydrochloric acid solution and mixed well, took 0.2 mL of the mixed solution in another test tube, added 2.5 mL phenol solution and 2.5 mL sodium hypochlorite solution in turn, mixed well, placed it in a 60 °C constant temperature water bath to react for 10 min, Then measured the absorbance value (OD) at the 560 nm wavelength, used the NH_4_Cl standard curve to calculate the NH_4_Cl concentration in the sample solution.

$$\mathrm{AN}\ \left(\%/\mathrm{DM}\right)=\mathrm{C}\ast 14\ast 1.8\ast 1{0}^4/\mathrm{W}\ast \mathrm{M}$$

Note: C represents the NH_4_Cl concentration in the sample solution (mg/mL).

M represents the NH_4_Cl relative molecular mass.

W represents the weight of 20 g sample (g).

(the standard curve line y = 1.5907x - 0.0087 R^2^ = 0.999 y: OD x = C)

### Microbial analysis

Microbial population on fresh samples was determined by the method of Cai [23]. Fresh samples (10 g) were put into a sterile glass bottle, and the concentration was diluted from 10^− 1^ to 10^−3^with sterile water for serial dilution. Lactic acid bacteria were counted on MRS agar medium (GCM188, Beijing Luqiao Technology Co., Ltd., Beijing, China), yeasts and molds were numbered on malt extract agar (021110, Huankai Microbial Technology Co., Ltd., Guangzhou, China). The microbial count was expressed as log_10_ cfu/g FM.

The DNA of bacteria in the silage sample was extracted by the method of Li et al. [19]. And the Phusion® high-fidelity PCR master mix (New England Biolabs) was used for the PCR reaction. 515F (5′-CCTACGGGAGGCAGCAG-3′) and 907R (5′-TTACCGCGGCTGCTGGC-3) were taken as primers to amplify the 16S rRNA gene. The PCR products were purified using Qiagen Gel Extraction Kit (Qiagen, Germany), and then sequenced at the paired ends (250 bp) of the Illumina MiSeq PE2500 platform at Novogene Company. All barcodes and primers were discarded to obtain high-quality sequences. After sequencing, trimmomatic was used to process the original sequence, and the PE reading was overlapped with FLASH (V 1.2.7) to assemble the final V3-V4 tag sequence. The valid label was generated by the method of Uchime (version 4.2.40). On the Usearch software platform (version 7.1), the Uparse method was used to assign the operational taxonomic unit (OTU) to the 16S rRNA at a cut-off level of 3%. According to the results of OTU, Mothur (version v.1.30.1) was used to generate the alpha diversity (Chao 1 and Shannon index).

### Statistical analysis

Data of silages was subjected to one-way analysis ANOVA. Significant differences between means were considered significant for *P* < 0.05 by Tukey’s Studentized range test. This analysis was performed using the SPSS 19.0 program.

## Results

### Chemical composition and microbial population of reed canary grass silage

The chemical composition of RCG silage was shown in Table 1. Compared with control, PLA decreased (*P* < 0.05) the WSC and ADF contents, and increased (*P* < 0.05) the CP content of RCG silage; LABi increased (*P* < 0.05) the WSC and CP contents of silage; PSB, PLA and LABi increased the count of LAB. In addition, yeasts were not observed in silage treated with PLA and PLA + LABi.

**Table 1:** Chemical composition and microbial population of RCG silage

Treatment	DM	WSC	CP	NDF	ADF	LAB	Yeasts
CK	27.74^b^	6.95^b^	8.29^b^	67.62	40.81^a^	6.0 ± 1.0	4.0 ± 0^a^
PSB	28.44^a^	6.96^b^	9.03^ab^	68.09	40.28^a^	6.7 ± 0.58	3.0 ± 0^a^
PLA	28.50^a^	5.29^c^	9.28^a^	69.47	38.81^b^	7.0 ± 0	No detected
LABi	28.66^a^	9.08^a^	9.35^a^	69.72	40.52^a^	7.0 ± 0	0.7 ± 1.15^c^
PLA + LABi	28.77^a^	7.25^b^	8.76^ab^	69.91	40.42^a^	7.0 ± 0	No detected
SEM	0.119	0.343	0.187	0.413	0.213	0.150	0.456

Silages treated without (CK) or with antimicrobial additive (PSB), phenyllactic acid (PLA), lactic acid inoculant (LABi) and PLA + LABi. ADF, acid detergent fiber; *CP* crude protein, *DM* dry matter, *FW* fresh wight, *LAB* lactic acid bacteria, *NDF* neutral detergent fiber, *SEM* stands for mean standard error. Values with different letters in the same rows are significantly different (*P* < 0.05)

As shown in Table 2, all additives reduced (*P* < 0.05) the lactic acid content of silage as compared to control. Silages treated with LABi and PLA + LABi had lower (*P* < 0.05) pH value, acetic acid content and ammonia-N ratio of total N.

**Table 2:** Fermentation quality of reed canary grass silage

Treatment	pH	Lactic acid	Acetic acid	Propionic acid	Butyric acid	Ammonia-N
		% DM				% total N
CK	6.60^a^	8.84^a^	2.06^a^	2.24^ab^	Not detected	7.18^a^
PSB	6.58^a^	5.78^b^	1.40^ab^	1.59^d^	0.32^c^	5.07^d^
PLA	6.51^a^	5.85^b^	1.39^ab^	2.46^a^	1.07^a^	6.25^ab^
LABi	5.29^b^	2.67^c^	0.69^bc^	2.01^bc^	0.75^b^	5.20^cd^
PLA + LABi	5.48^b^	3.35^c^	Not detected	1.90^cd^	1.04^a^	6.10^bc^
SEM	0.164	0.624	0.213	0.429	0.113	0.132

Silage treated without (CK) or with antimicrobial additive (PSB), phenyllactic acid (PLA), lactic acid inoculant (LABi) and PLA + LABi. SEM stands for mean standard error. Values with different letters in the same rows are significantly different (*P* < 0.05)

### Bacterial composition of reed canary grass silage

The bacterial alpha diversity of silage is shown in Table 3. Compared with control, PLA and LABi reduced (*P* < 0.05) the bacterial Chao1, observed OTUs and Simpson indices in silage. In addition, PLA and LABi significantly reduced (*P* < 0.05) the observed OTUs and Simpson indices as compared with PSB.

**Table 3:** Bacterial alpha diversity of reed canary grass silage

Treatment	Observed OTUs	Chao 1	Simpson	PD whole tree	Goods coverage
CK	39^a^	58.33^a^	0.51^a^	3.37^a^	0.996^b^
PSB	33^a^	46.18^ab^	0.61^a^	2.79^a^	0,997^ab^
PLA	26^b^	28.98^bc^	0.24^b^	2.04^ab^	0.998^ab^
LABi	15^b^	18.72^c^	0.23^b^	1.21^b^	0.999^a^
PLA + LABi	37^a^	44.48^ab^	0.71^a^	3.04^a^	0.997^ab^
SEM	4.640	6.853	0.101	0.381	0.001

Silage treated without (CK) or with antimicrobial additive (PSB), phenyllactic acid (PLA), lactic acid inoculant (LABi) and PLA + LABi. SEM stands for mean standard error. Values with different letters in the same rows are significantly different (*P* < 0.05)

**Fig. 1:**
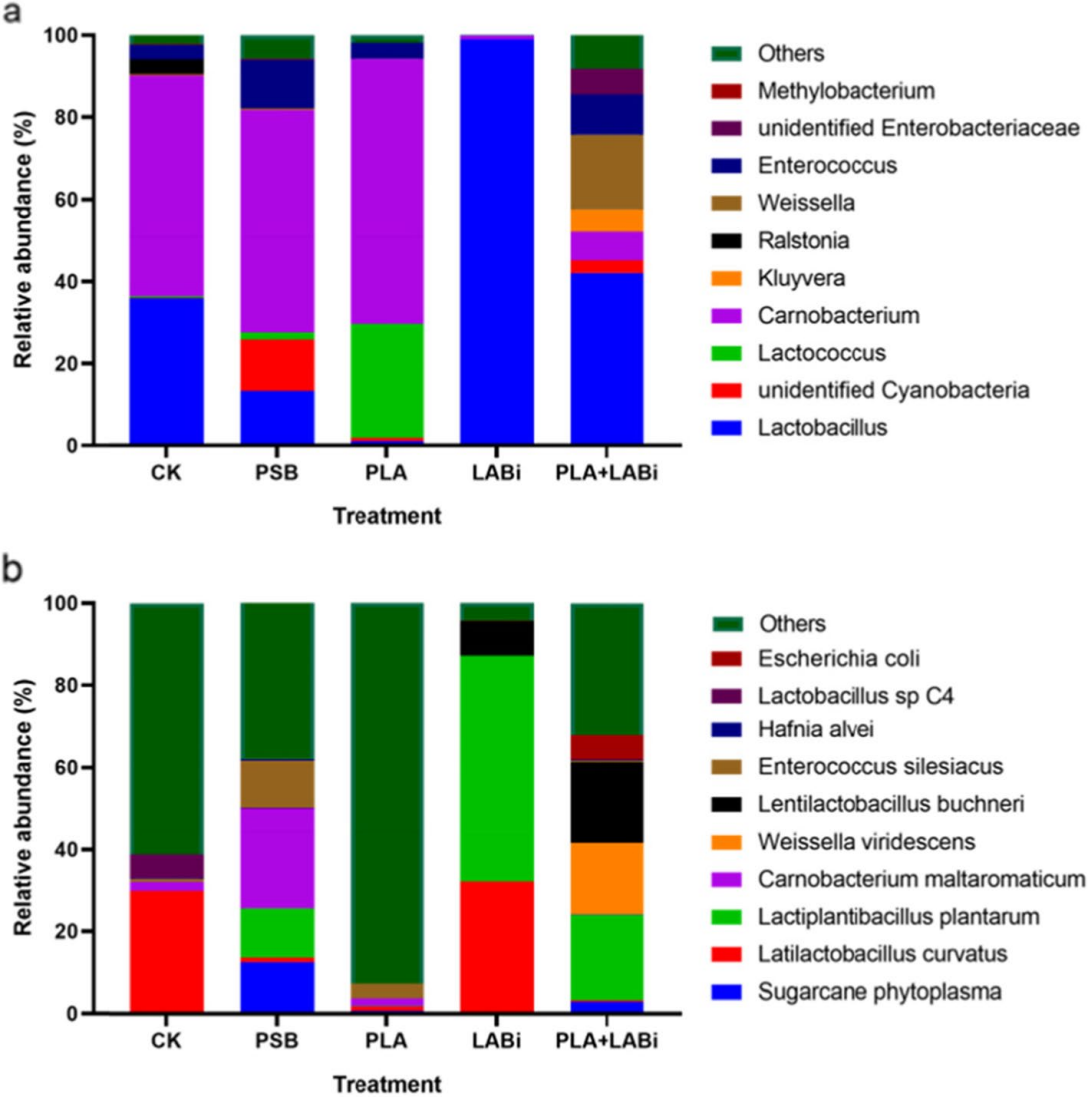
Bacterial community composition of reed canary grass silage at genus (**a**) and specie (**b**) levels. Silage treated without (CK) or with antimicrobial additive (PSB), phenyllactic acid (PLA), lactic acid inoculant (LABi) and PLA + LABi

The bacterial community of silage was shown in Fig. 1. At the genus level, *Carnobacterium* (54.94%) and *Lactobacillus* (35.45%) were observed in control silage (Fig. 1a). PLA increased the relative abundance of *Lactococcus*, while reduced the relative abundance of *Lactobacillus* in silage in relative to control. *Lactobacillus* was the dominant genus in LABi silage. PLA + LABi silage showed a higher abundance of *Lactobacillus* (80.36%). At the species level, *L. curvatus* (29.83%) was observed in control silage. PSB silage showed high abundance of *L. plantarum* and *Sugarcane phytoplasma* (Fig.1b). Low dominance of *Lactobacillus* species (*L. curvatus* and *L. buchneri*) were observed in PLA silage. *Lactobacillus* species (*L. plantarum* and *L. curvatus*) became a dominant flora in LABi silage. PLA + LABi silage increased the relative abundance of *L. plantarum* and *L. buchneri* as compared with control silage.

**Fig. 2:**
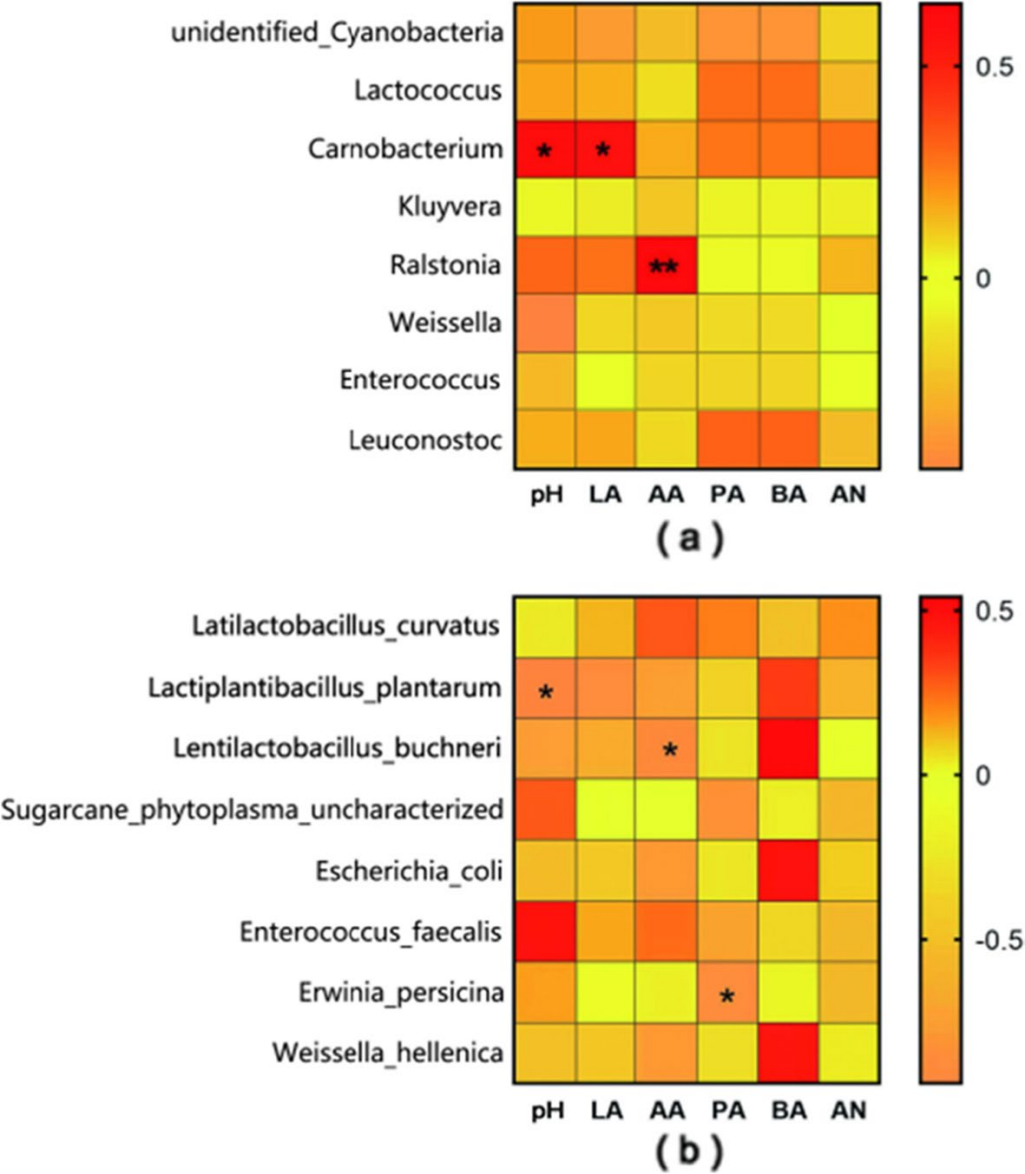
Fermentation factors affecting bacterial genus level (**a**) and species level (**b**) community composition of reed canary grass silage. LA, lactic acid; AA, acetic acid; PA, propionic acid; BA, butyric acid; AN, ammonia-N. * Significant at *P* < 0.05; ** Significant at *P* < 0.01

The changes in fermentation characteristics during ensiling are inseparable from the metabolism of microbial flora. As shown in Fig. 2, *Carnobacterium* was positively correlated (*P <* 0.05) with pH value and lactic acid content in silage. *Ralstonia* was highly positively correlated (*P <* 0.01) with acetic acid content. The relative abundance of *L. plantarum* was negatively correlated (*P <* 0.05) with pH value. The relative abundance of *L. curvatus* was negatively correlated (*P <* 0.05) with acetic acid content. The count of *Erwinia persicina* was negatively correlated with propionic acid content (*P* < 0.05).

## Discussion

### Chemical composition and microbial population of reed canary grass silage

The CP is an important nutritional indictor in silage. In this study, higher CP content was observed in PLA and LABi silages. PLA can inhibit the growth of yeasts to reduce the consumption of nutrients [24]. In addition, LAB can secrete antibacterial substances to inhibit the growth of harmful microorganisms [25]. PLA reduced ADF content in RCG silage. Similar results were from Li et al. who reported that the fiber content of silage reduced as the ratio of crude protein increased [26]. Therefore, to some extent, PLA increased the nutritional value of the silage. The beneficial bacteria, LAB, played a significant part in the silage fermentation process [27]. Under anaerobic conditions, LAB can reduce pH value by generating organic acids (mainly lactic acid) and secrete antibacterial substances to inhibit the growth of undesirable microorganisms [19, 23]. In this study, applications of PSB, PLA and LABi at ensiling promoted the growth of LAB in silage. In this study, as expected, PLA alone or in combine with LABi decreased the count of yeasts in silage.

The pH value is one of the important indicators for evaluating the quality of silage [28]. Too high pH value will promote the growth of acid-intolerant and harmful microorganisms such as *Clostridium*, making the silage fermentation bad. Too low pH value will inhibit the growth of LAB and affect the fermentation quality. In this study, inoculation of exogenous LAB reduced pH value in silage. Similar results were from Li et al. who reported that the inoculation of exogenous LAB (*L. plantarum* and *L. buchneri*) had a positive effect on the fermentation quality of RCG silage [19]. It will promote LAB to produce lactic acid and acetic acid for reducing the pH value in silage. In this study, PLA + LABi silage had a lower pH value than control silage, which may be related to the combined effect of phenyllactic acid and exogenous LAB. Low lactic acid content was observed in PSB and PLA-treated silages. A high pH value reduced the antibacterial effects of potassium sorbate and sodium benzoate. A study from Weinberg & Muck showed that potassium sorbate and sodium benzoate had a poor antibacterial effect inhibiting the growth of undesirable microorganisms, such as yeasts and molds at the pH value > 5 [29]. Previous studies showed that PLA could inhibit the fermentation of LAB [30]. Meanwhile, exogenous LAB produced biological antagonism against inherent LAB adhered to the raw materials [31]. Acetic acid showed the second highest concentration of organic acid in silage. The appropriate concentration of acetic acid was beneficial to the fermentation of silage. Low acetic acid content may increase the potential of aerobic deterioration in silage [8]. In this study, treatments with LABi and PLA + LABi had low acetic acid content. This was attributed to the dry matter content [8]. In this study, higher butyric acid content was observed in PLA silage. This may be related to that both Clostridia and plant proteolytic enzymes were active at the pH value > 5 [32]. This also indicated that ammonia-N was not significantly reduced in PLA silage. Ammonia-N is also an important index for evaluating the quality of silage, which reflected the activity of plant proteases or the degree of protein degradation based on clostridial fermentation [33]. In this study, treatments with PSB, LABi and PLA + LABi had lower ammonia-N contents than the control, which indicated that PSB, LABi and PLA + LABi inhibited the growth and propagation of harmful microorganisms in silage, thereby reducing the degradation of protein.

### Microbial composition of reed canary grass silage

In this study, the goods coverage was > 0.99, indicating that sequencing depth covered the microbial composition in silage. PLA-treated silage had lower Chao1, observed OTUs and Simpson indices, which suggested that PLA reduced the microbial diversity in silage. The possible reason was that LAB became the dominant group in PLA silage and inhibited the growth of other bacteria [34]. However, silage treated with PLA + LABi showed a higher diversity as compared with control. This may be because the organic acid content was too low to inhibit bacteria in PLA + LABi silage. Moreover, we found that PLA silage had lower observed OTUs and Simpson indexes than PSB silage, which may be attributed to the growth and propagation of LAB in silage. Previous studies showed that LAB can produce some antibacterial substances in the metabolic process to effectively inhibit the growth of harmful microorganisms, such as yeasts, molds and clostridiums [25].

*Carnobacterium* and *Lactobacillus* were dominant genera in control silage. Genus of *Carnobacterium* was considered as harmful bacteria, which may cause the poor quality of silage [35]. PLA silage increased the abundance of *Carnobacterium* and *Lactococcus.* Furthermore, PLA silage decreased the relative abundance of *Lactobacillus.* This may be attributed to the high pH value being observed in PLA silage. Previous studies revealed that it was more conducive to the growth of *Carnobacterium* and *Lactococcus* in a high-pH environment, but was not good for the growth of more acid-tolerant *Lactobacillus* [36–38]. *Lactococcus* is one of the ideal functional bacteria in silage fermentation process. During fermentation, they secrete lactic acid to lower the pH value, thereby improving the quality of silage [39, 40]. Therefore, they played an important role in improving the quality of silage. In addition, *Lactobacillus* became a dominant genus in LABi-treated silage, which was consistent to the results of Bai et al. who reported that the inoculation of LAB can increase the abundance of *Lactobacillus* in silage [34]. The dominant genus was *Lactobacillus* in PLA + LABi silage, which may be attributable to the fact that PLA and LAB had the joint broad-spectrum antibacterial effect for inhibiting the growth of undesirable microorganisms [41].

*L. curvatus*, as a facultative hetero-fermentative LAB, dominated in control silage. Similar results were reported by Terán et al. [42]. In addition, it had better lactic acid productivity than *L. plantarum* under low temperature conditions, which may indicate the high lactic acid content in control silage [43]. Previous studies showed that potassium sorbate and other inorganic salt additives can generate corresponding organic acids when dissolved in water. These organic acids can penetrate into the cell membranes of LAB, destroying their biological activity and inhibiting their growth [44]. PLA silage decreased the abundance of *Sugarcane phytoplasma* (uncharacterized), *L. plantarum* and *L. curvatus*, which indicated that PLA can inhibit both undesirable microorganisms and LAB due to the high concentration of propionic acid in PLA silage, which can inhibit the activity of microorganisms including LAB [19]. However, a study from Jung et al. showed that PLA can promote the growth of LAB. In addition, Wu’s findings showed that PLA had no effect on the growth of LAB [15]. Therefore, the effect of PLA on the growth of LAB needed to be further studied. For example, we can explore the mechanism of action of PLA on the growth of LAB through macro-genomics and metabonomics.

*Carnobacterium* is gram-positive bacteria, which is able to produce lactic acid from glucose and closely related to *Enterococcus* [36]. However, it is often found in poor-fermentation silage with a high pH value [45]. Many studies proved that *L. plantarum* can effectively reduce the pH value [46, 47]. This suggested the relationship between *L. plantarum* and pH value. Previous studies showed that bacteria of the genus *Ralstonia* can secrete short-chain fatty acids to alleviate the inflammatory response in intestine [48]. Acetic acid is a kind of short-chain fatty acid, so the increased count of *Ralstonia* can increase acetic acid content. Species of *L. buchneri* is a hetero-fermentative LAB, which is able to metabolize 1,2-propanediol to produce propionic acid [49]. Propionic acid can inhibit the activity of acetic acid-producing microorganisms, thereby reducing acetic acid content [50]. It is well known that propionic acid can inhibit the growth of undesirable microorganisms, such as *Erwiniapersicina* [51, 52].

## Conclusion

Application of PLA during ensiling can improve the nutritional quality of RCG silage. PLA promoted the growth of LAB and reduced the bacterial alpha diversity in silage. The combination of PLA and LABi increased the relative abundance of *Lactobacillus* and shaped the bacterial community of silage. In conclusion, this study confirmed that PLA, as an antifungal silage additive, exerted a good effect in improving the quality of silage. However, a further study will conduct to explore the effect of PLA-producing bacteria on silage quality.

## Data Availability

The datasets generated and/or analyzed during the current study are available in the: https://pan.baidu.com/s/1knpPlyWa_QB5dRpAHlzNJw and extraction code: 1111.

## Abbreviations

DM: Dry matter
WSC: Water-soluble carbohydrates
CP: Crude protein
NDF: Neutral detergent fiber
ADF: Acid detergent fiber
LAB: Lactic acid bacteria
LA: Lactic acid
AA: Acetic acid
PA: Propionic acid
BA: Butyric acid
AN: Ammonia nitrogen
